# Association between pretransfer cleavage-stage blastomere dynamics and pregnancy outcomes in fresh single embryo transfer cycles: a retrospective cohort study

**DOI:** 10.3389/fendo.2025.1672664

**Published:** 2025-09-30

**Authors:** Qingkai Wang, Shuangshuang Geng, Hansheng Feng, Bin Zhang, Kai Deng, Zimo Zhao, Jinfeng Xu, Haiye Wang, Jing Wang, Weimin Yang, Liyi Cai

**Affiliations:** ^1^ Department of Reproductive Medicine, Hebei Reproductive Maternity Hospital, Shijiazhuang, Hebei, China; ^2^ Shi Jiazhuang Technology Innovation Center of Precision Prevention and Control of Birth Defects, Shijiazhuang, Hebei, China; ^3^ State Key Laboratory of Animal Biotech, Breeding Frontiers Science Center for Molecular Design Breeding, College of Biological Sciences, China Agricultural University, Beijing, China

**Keywords:** single embryo transfer, fresh transfer, day 3 embryo, blastomere number, pregnancy outcome

## Abstract

**Objective:**

To evaluate the impact of embryo blastomere cell number and dynamic changes in cell number during the morning of embryo transfer day (7:00–11:00) on clinical pregnancy outcomes in fresh single embryo transfer (SET) cycles.

**Methods:**

A retrospective cohort study was performed, including 561 fresh SET cycles conducted between January 2022 and June 2024. Cycles were categorized into four groups based on embryo blastomere count before transfer: ≤7-cell, 8-cell, 9–10-cell, and ≥11-cell groups. We analyzed the relationship between the increase in blastomere number observed from 7:00 a.m. to 11:00 a.m. on the day of transfer and clinical pregnancy outcomes. Multivariate logistic regression analysis was utilized to assess the influence of various factors on clinical pregnancy and live birth rates.

**Results:**

Clinical pregnancy rates significantly differed among the ≤7-cell, 8-cell, 9–10-cell, and ≥11-cell groups (10.64%, 36.69%, 42.31%, and 46.32%, respectively; P = 0.004). Live birth and biochemical pregnancy rates exhibited a similar increasing trend with higher cell numbers (P = 0.001), whereas early miscarriage rates showed no significant differences among groups (P = 0.157). In the 9–10-cell group, embryos that exhibited an increase in blastomere number had significantly higher clinical pregnancy rates (50% vs. 23.68%, P = 0.006) and live birth rates (41.30% vs. 15.79%, P = 0.005). No significant differences were observed in the ≤7-cell and 8-cell groups (P > 0.05). Multivariate logistic regression analysis demonstrated that increased endometrial thickness significantly improved clinical pregnancy likelihood (P = 0.034), whereas lower blastomere number (≤7-cell) significantly reduced clinical pregnancy rates (P = 0.002).

**Conclusion:**

A higher embryo blastomere count before transfer is significantly associated with improved clinical pregnancy and live birth outcomes in fresh SET cycles. Short-term increases in blastomere number on the morning of transfer day may reflect superior embryo developmental potential.

## Introduction

Single embryo transfer (SET) has been widely adopted in assisted reproductive technology (ART). Previous studies have shown that fresh day 3 (D3) SET effectively reduces multiple pregnancies and improves overall pregnancy outcomes (1, 2), thereby enhancing perinatal safety. However, accurately selecting high-quality embryos to achieve optimal pregnancy outcomes remains a critical issue in ART.

Currently, routine clinical indicators used for embryo quality assessment include morphological grading, blastomere number, and fragmentation rate (3, 4). Among these, the number of blastomeres is considered an essential parameter reflecting embryonic developmental potential (5–7). According to the Istanbul consensus, embryos reaching the 8-cell stage on D3 post-fertilization are deemed to exhibit ideal developmental rates (8, 9). Nevertheless, recent findings from Tian et al. (10) indicated that embryos exceeding the 8-cell stage on D3 could be associated with higher live birth rates (LBR) in fresh transfer cycles. Additionally, several studies have shown that increased blastomere numbers on D3 correlate positively with higher-quality blastocyst formation rates and improved clinical pregnancy outcomes following single blastocyst transfer on day 5 (11–14). Conversely, other research suggests an increased blastomere number at the cleavage stage may correlate with higher rates of embryonic aneuploidy (15, 16). However, these studies primarily focused on a single observation window around 68 ± 1 hours post-fertilization (i.e., the morning of D3), with very limited data regarding embryo development during the short interval immediately preceding embryo transfer.

In clinical practice, embryo quality is typically evaluated approximately 68 hours after fertilization or intracytoplasmic sperm injection (ICSI), most commonly at around 7:00 a.m. on D3. However, in real-world settings, embryo transfer is often scheduled around 11:00 a.m. During this interval (7:00 to 11:00 a.m.), embryos may continue to cleave, leading to an increased blastomere count. The clinical relevance of this short-term cleavage extension remains poorly studied, and its effect on pregnancy outcomes has yet to be clearly established.

Accordingly, this study adopted a retrospective cohort design to investigate the relationship between embryo blastomere number and short-term dynamic changes during the morning interval prior to transfer in fresh single embryo transfer (SET) cycles. The objective was to generate new insights into optimizing clinical pregnancy outcomes and to provide practical, broadly applicable, and low-threshold auxiliary criteria to support embryo selection.

## Methods

### Study population

A retrospective analysis was conducted on patients who underwent fresh SET at the authors’center between January 2022 and June 2024. Cycles involving the transfer of two embryos or with missing data were excluded. Based on blastomere count prior to embryo transfer, patients were divided into four groups: ≤7-cell, 8-cell, 9–10-cell, and ≥11-cell groups. Additionally, dynamic changes in blastomere number were recorded on the morning of the transfer day, between 7:00 and 11:00 a.m.

### Ovulation stimulation and embryo culture

The ovarian stimulation protocols were individualized based on each patient’s ovarian reserve, ovarian response, and hormone levels, with either a long protocol or a GnRH antagonist protocol being applied. During treatment, gonadotropin (Gn) doses were adjusted dynamically according to serum hormone levels and follicular development. When follicles reached the criteria for triggering final oocyte maturation, 6000–10000 IU of human chorionic gonadotropin (hCG), or a combination of 0.1–0.2 mg of triptorelin acetate (Dabijia) and 2000–4000 IU hCG, was administered. Oocyte retrieval was performed 37 hours after trigger under ultrasound guidance. The choice of fertilization method was based on clinical indications. Conventional *in vitro* fertilization was used for patients with normal semen parameters, whereas ICSI was applied for patients with severe oligozoospermia, asthenozoospermia, teratozoospermia, epididymal or testicular sperm retrieval, previous fertilization failure, or markedly reduced fertilization rates, as determined by the attending clinicians. Embryo culture was carried out using commercial media from the Vitrolife series (Vitrolife, Sweden; Catalog Nos. 10086, 10127, 10131). To maintain a stable culture environment, all dishes were overlaid with 100% mineral oil (SAGE Laboratories, Denmark; Catalog No. ART-4008), and culture conditions were strictly controlled: temperature was maintained at 37°C, with a gas mixture of 6% CO_2_ and 5% O_2_ to optimize embryo development. This study assessed the embryo cell count at 68 ± 1 hours post-fertilization, while also recording other embryonic morphological indicators, including blastomere symmetry, embryo fragmentation rate, and the presence of multinucleation or vacuolation. The cell count was re-evaluated before embryo transfer. To minimize inter-observer variability, each embryo was graded by two embryologists for every patient.

### Embryo transfer

For patients undergoing fresh embryo transfer, luteal phase support was initiated on the day of oocyte retrieval with daily intramuscular injections of progesterone (40 mg/day; Xianju Pharma, China). On D3 post-retrieval, cleavage-stage embryo transfer was performed under transabdominal ultrasound guidance. After transfer, luteal support was continued using vaginal progesterone gel (Crinone, Merck Serono, Switzerland; 90 mg/day) in combination with oral dydrogesterone (Duphaston, Abbott Biologicals, USA; 20 mg/day), and maintained until 8–10 weeks of gestation upon confirmation of clinical pregnancy.

### Statistical methods

All statistical analyses were performed using SPSS version 25.0. Continuous variables with normal distribution were expressed as mean ± standard deviation ( $\bar{\text{x}}$ ± s) and compared using one-way ANOVA, while non-normally distributed continuous variables were expressed as median and interquartile range [Md (Q1, Q3)] and compared using the Kruskal–Wallis H test. When overall significance was observed with more than two groups, *post hoc* pairwise comparisons were performed with Bonferroni adjustment, and adjusted P-values (P_adj) ≤ 0.05 were considered statistically significant. Categorical variables were analyzed using Pearson’s chi-square test (χ²). In addition, multivariate logistic regression was used to identify factors associated with clinical pregnancy and live birth. A two-tailed P-value < 0.05 was considered statistically significant.

### Ethics approval

This study was approved by the Ethics Review Committee of Hebei Maternity Hospital and qualifies for exemption from informed consent requirements; patient information was anonymized and de-identified prior to analysis.

## Results

**Figure 1:**
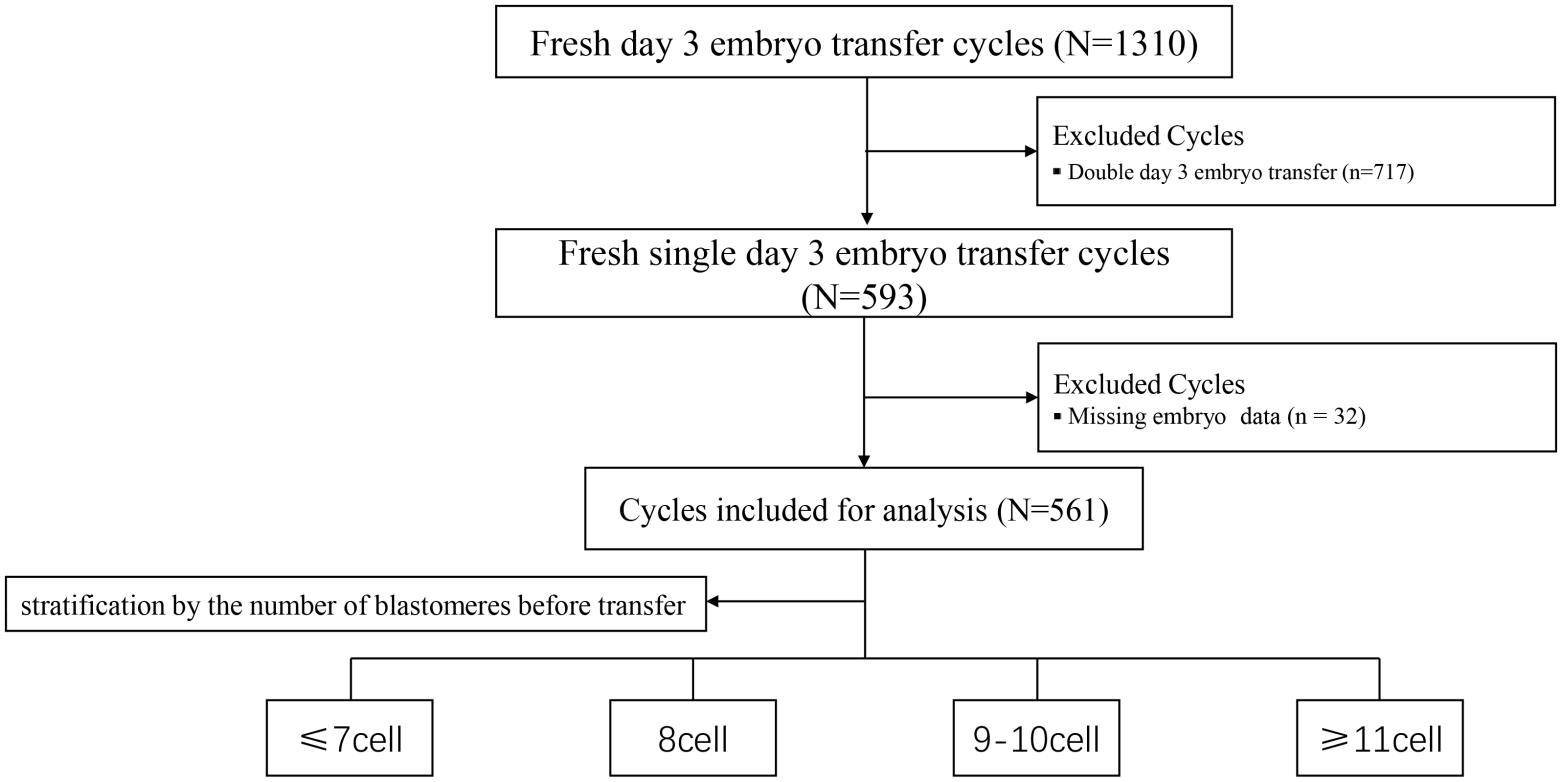
Flowchart of data selection process for analysis.

A total of 561 participants were enrolled and stratified into four groups based on blastomere count prior to embryo transfer: ≤7-cell, 8-cell, 9–10-cell, and ≥11-cell groups. The study flowchart is presented in **Figure 1**, and baseline demographic characteristics are summarized in **Table 1**. No significant differences were observed among groups in maternal age, body mass index (BMI), infertility duration, endometrial thickness, infertility type, luteinizing hormone (LH), or estradiol (E2) levels, except for follicle-stimulating hormone (FSH) and anti-Müllerian hormone (AMH) levels (both P < 0.05). Bonferroni-adjusted *post hoc* comparisons confirmed that the ≤7-cell group had significantly higher FSH levels and lower AMH levels compared with the other groups (P_adj < 0.05).

**Table 1:** Baseline demographic and clinical characteristics of patients stratified by blastomere number prior to transfer.

Variable	≤7cell	8cell	9-10cell	≥11cell	*P*
No. of patients	47	248	130	136	
Age of women (years)	33.46 ± 5.16	32.02 ± 4.48	31.52 ± 4.82	31.46 ± 4.71	0.057
BMI (kg/m²)	24.74 ± 4.62	23.85 ± 3.99	24.31 ± 4.26	24.59 ± 4.36	0.288
Duration of infertility (years)	2.00 (1.00,5.00)	3.00 (1.00,4.00)	2.50 (1.00,5.00)	3.00 (1.00,4.75)	0.723
Endometrial thickness (mm)	10.00 (8.98,12.33)	10.40 (9.00,11.80)	10.65 (9.00,12.03)	10.50 (9.23,12.60)	0.619
Fertilization method					0.077
IVF	30 (63.83)	197 (79.44)	110 (84.62)	108 (79.41)	
ICSI	15 (31.91)	41 (16.53)	19 (14.62)	24 (17.65)	
RICSI	2 (4.26)	10 (4.03)	1 (0.76)	4 (2.94)	
Infertility causes, n (%)					0.565
Female reason	38 (80.85)	174 (70.16)	100 (76.92)	105 (77.21)	
Male reason	4 (8.51)	33 (13.31)	13 (10.00)	17 (12.50)	
Reasons of both sides	2 (4.26)	10 (4.03)	2(1.54)	2(1.47)	
Unknown reason	3 (6.38)	31 (12.5)	15 (11.54)	12 (8.82)	
Type of Infertility					0.120
Primary	28 (59.60)	107 (43.10)	59 (45.40)	74 (54.40)	
Secondary	19 (40.40)	141 (56.90)	71(54.60)	62 (45.60)	
Basal hormone profile					
FSH (mIU/mL)	8.14 (6.25,11.63)^a^	7.22 (5.95,8.92)^b^	7.08 (5.72,8.48)^b^	6.65 (5.55,8.29)^b^	0.003
LH (mIU/mL)	3.75 (2.52,5.90)	3.95 (2.73,5.90)	3.72 (2.77,5.11)	4.23 (3.07 ,5.65)	0.737
E2 (pg/mL)	28.29 (21.88,46.06)	34.70 (25.16,47.36)	36.08 (26.76,47.76)	33.31 (24.96,44.70)	0.500
AMH (ng/mL)	1.33 (0.34,2.60)^a^	2.37(1.44,3.99)^b^	2.60(1.40,4.31)^b^	3.09(2.04,4.68)^b^	<0.001

BMI, body mass index; FSH, follicle-stimulating hormone; LH, luteinizing hormone; E2, estradiol; AMH, anti-Müllerian hormone.

Values are presented as mean ± standard deviation or median (interquartile range) for continuous variables and as number (percentage) for categorical variables. P-values were calculated using one-way ANOVA, Kruskal–Wallis H test, or χ² test, as appropriate. Post hoc pairwise comparisons were performed with Bonferroni adjustment. Within each row, values that do not share the same superscript letter (a, b) differ significantly (P ≤ 0.05).

Pregnancy outcomes for each group are summarized in **Table 2**. The LBR differed significantly among groups: 4.26% (≤7-cells), 27.82% (8-cells), 33.85% (9–10-cells), and 38.97% (≥11-cells) (P = 0.001). Similarly, both the hCG-positive rate and clinical pregnancy rate (CPR) increased significantly with higher blastomere counts (P = 0.004 and P = 0.001, respectively). Although the early miscarriage rate was highest in the ≤7-cell group (40.00%), the differences among groups were not statistically significant (P = 0.157). Bonferroni-adjusted *post hoc* comparisons indicated that the ≤7-cell group had significantly lower CPR and LBR compared with the ≥8-cell groups (P_adj < 0.05). In a subgroup analysis of patients aged ≤35 years (**Supplementary Table 1**), LBR showed a significant positive trend with increasing blastomere number—6.7%, 29.9%, 31.8%, and 41.7% across the respective groups (P = 0.003). CPR also increased significantly with blastomere count (P = 0.009), and Bonferroni-adjusted *post hoc* comparisons confirmed that the ≤7-cell group had significantly lower CPR and LBR compared with the ≥8-cell groups (P_adj < 0.05). **Supplementary Table 3** compares the clinical outcomes between the 8-cell group (n=248) and the >8-cell group (n=266). The results showed that the >8-cell group had higher hCG-positive (54.14% vs. 47.58%) and CPR (44.36% vs. 36.69%) compared with the 8-cell group, although these differences did not reach statistical significance (P = 0.162 and P = 0.093, respectively). In contrast, the LBR was significantly higher in the >8-cell group than in the 8-cell group (36.47% vs. 27.82%, P = 0.046).

**Table 2:** Pregnancy outcomes stratified by number of blastomeres prior to transfer.

Variables	≤7cell n=47	8cell n=248	9-10cell n=130	≥11cell n=136	*P*
HCG positive rate, n (%)	12 (25.53)^a^	118 (47.58)^b^	72 (55.38)^b^	72 (52.94)^b^	0.004
Clinical pregnancy, n (%)	5 (10.64)^a^	91 (36.69)^b^	55 (42.31)^b^	63 (46.32)^b^	0.001
Early miscarriage, n (%)	2 (40.00)	15 (16.48)	8 (14.55)	5 (7.94)	0.157
Live birth, n (%)	2 (4.26)^a^	69 (27.82)^b^	44 (33.85)^b^	53 (38.97)^b^	0.001

Values are presented as number (percentage). P-values were calculated using χ² test. Post hoc pairwise comparisons were performed with Tukey’s test and Bonferroni adjustment. Within each row, values that do not share the same superscript letter (a, b) differ significantly (P ≤ 0.05).

Associations between dynamic changes in embryo blastomere count on transfer day (7:00 to 11:00 a.m.) and pregnancy outcomes across baseline blastomere-count groups are presented in **Table 3**. In the 9–10-cell group, embryos exhibiting dynamic blastomere increases showed significantly higher CPR (50.00% vs. 23.68%; P = 0.006) and LBR (41.30% vs. 15.79%; P = 0.005) compared with embryos without increases. In the 8-cell and ≥11-cell groups, embryos with increased blastomere counts also had numerically higher CPR and LBR, but these differences were not statistically significant (P > 0.05). In the ≤7-cell group, no comparative analysis was conducted due to limited sample size and absence of dynamic increases.

**Table 3:** Blastomere dynamics and pregnancy outcomes by cell count group.

	Increase in cell number (Y/N)	Clinical pregnancy, n (%)	*P*	Live birth, n (%)	*P*
≤7cell	Y	--	--	--	--
	N	11.36% (5/44)		4.55% (2/44)	
8cell	Y	29.42% (5/17)	0.519	17.65% (3/17)	0.332
	N	37.22% (86/231)		28.57% (66/231)	
9-10cell	Y	50% (46/92)	0.006	41.30 (38/92)	0.005
	N	23.68% (9/38)		15.79 (6/38)	
≥11cell	Y	48.45% (47/97)	0.345	40.21% (39/97)	0.520
	N	39.47% (15/38)		34.21% (13/38)	

Y, Yes (presence of blastomere number increase); N, No (no increase in blastomere number).

**Supplementary Table 2** explores the relationship between short-term blastomere increase (7:00 to 11:00 a.m. on transfer day) and pregnancy outcomes. Embryos demonstrating increases of 1–2 or ≥3 blastomeres exhibited significantly higher CPRs (46.40% and 47.67% vs. 32.86%; P = 0.004) and LBR (38.40% and 38.37% vs. 24.86%; P = 0.003), compared with embryos without increases. Similarly, biochemical pregnancy rates increased with blastomere number (44.25%, 58.40%, and 53.48%; P = 0.016). However, early miscarriage rates did not differ significantly across the groups (15.65%, 15.52%, and 7.32%; P = 0.485). These differences remained significant after Bonferroni-adjusted *post hoc* comparisons, with embryos showing blastomere increases (≥1) achieving superior CPR and LBR compared with those without increases (P_adj < 0.05).

Multivariate logistic regression was conducted to control for potential confounding factors, including maternal age, BMI, infertility type, infertility duration, AMH, endometrial thickness, embryo blastomere count, and dynamic blastomere increases; detailed results are presented in **Table 4**. After adjusting for these variables, the CPR was significantly lower in the ≤7-cell group compared to the 8-cell group (aOR=0.21, 95% CI: 0.08–0.57; P = 0.002), suggesting that a lower blastomere count negatively impacts pregnancy outcomes. Although CPRs were marginally higher in the ≥11-cell (aOR=1.00, P = 0.986) and 9–10-cell groups (aOR=0.90, P = 0.710), differences were not statistically significant. Additionally, embryos with dynamic increases in blastomere count demonstrated a trend towards improved clinical pregnancy (aOR=1.49, 95% CI: 0.91–2.43; P = 0.109), although this did not reach statistical significance. Importantly, increased endometrial thickness on the day of transfer was positively associated with clinical pregnancy (aOR=1.10; P = 0.034), serving as an independent predictor.

**Table 4:** Multivariate logistic regression analysis of factors influencing clinical pregnancy.

Variable	OR	95% CI	*P*
Male age (years)	1.01	0.95, 1.07	0.829
Type of infertility			
Secondary infertility	—	—	
Primary infertility	0.96	0.65, 1.42	0.855
Duration of infertility (years)	0.95	0.88, 1.02	0.138
BMI (kg/m²)	1.03	0.99, 1.08	0.130
AMH (ng/mL)	1.03	0.94, 1.12	0.542
Number of oocytes retrieved	0.98	0.94, 1.03	0.449
Endometrial thickness on transfer day (mm)	1.10	1.01, 1.19	0.034
Increase in blastomere number			
No	—	—	
Yes	1.49	0.91, 2.43	0.109
Blastomere cell number group			
8cell	—	—	
≤7	0.21	0.08, 0.57	0.002
9-10cell	0.90	0.52, 1.56	0.710
≥10cell	1.00	0.58, 1.74	0.986

BMI, body mass index; AMH, anti-Müllerian hormone.

Neonatal outcomes across groups are described in **Table 5**. There were no significant differences among groups regarding neonatal sex distribution (P = 0.783). Birth weight, birth length, and gestational age also did not differ significantly among the embryo blastomere-count groups (all P > 0.05). Similarly, no significant differences were observed in the incidence rates of low birth weight, macrosomia, or preterm birth across groups.

**Table 5:** Neonatal outcomes by blastomere count group.

Variables	≤7cell n=47	8cell n=248	9-10cell n=130	≥11cell n=136	*P*
Gender, n (%)					0.783
Male	1 (50.00)	38 (55.07)	20 (45.45)	25 (47.17)	
Female	1 (50.00)	31 (44.93)	24 (54.55)	28 (52.83)	
Low-Weight n(%) < 2500 g, n (%)	1 (50.00)	4 (5.80)	2 (4.55)	5 (9.43)	0.086
Excess weight n(%) ≥ 4000 g, n (%)	0 (0.00)	4 (5.80)	1(2.27)	5 (9.43)	0.505
Preterm birth, n (%)	0 (0.00)	6 (8.70)	2 (4.55)	8 (15.10)	0.329
Singleton birth height(cm), mean ± SD	49.56 ± 2.72	49.00 ± 1.41	49.78 ± 2.02	49.55 ± 3.07	0.627
Singleton birth weight(kg), mean ± SD	2652.50 ± 915.70	3193.85 ± 501.96	3203.02 ± 419.91	3251.94 ± 577.00	0.613

## Discussions

This study investigated how the number of blastomeres and their short-term dynamic changes before embryo transfer influence clinical pregnancy outcomes in fresh SET cycles. Unlike previous research that mainly relied on static observations at 68 ± 1 hours post-fertilization (the morning of D3), our study uniquely emphasized the importance of observing dynamic changes in blastomere number within a brief window before embryo transfer, yielding clinically meaningful results.

Previous studies suggest that embryos reaching the 8-cell stage or dividing more rapidly by approximately 68 ± 1 hours post-fertilization exhibit superior implantation potential compared with embryos with slower division rates (17–19). In this context, our findings indicated that a higher blastomere count prior to transfer was positively associated with improved clinical pregnancy and live birth rates, with embryos containing ≥11-cells achieving the best outcomes. This suggests that rapid cleavage may serve as an important indicator of enhanced embryonic developmental potential. These results are consistent with the findings reported by Li et al. (20) and others (21, 22). Because the sample size of the ≤7-cell group was relatively small, which may have influenced the statistical stability, we further compared the clinical outcomes between the 8-cell and >8-cell group. The results showed that the live birth rate in the 8-cell group was significantly lower than that in the >8-cell group, suggesting that using the “8-cell” stage alone as the optimal D3 standard for embryo quality may have limitations. A potential explanation is that an increased number of blastomeres is generally associated with stronger developmental potential. Previous research has shown that embryos undergoing rapid division on D3 exhibit higher blastocyst formation rates (11, 15). Luna et al. (18) further indicated that embryos with rapid cleavage (≥10 cells) are more likely to become high-quality blastocysts compared with embryos with moderate cleavage. To mitigate age-related bias, we conducted subgroup analyses for patients aged ≤35 years, confirming that rapidly dividing embryos indeed yielded superior clinical outcomes.

Importantly, this study was the first to systematically explore the clinical significance of short-term dynamic increases in blastomere number observed on the morning of the embryo transfer day (7:00–11:00 a.m.). We compared blastomere counts at two distinct observation points: the traditional time point at approximately 68 ± 1 hours post-fertilization (around 7:00 a.m. on D3), and shortly before embryo transfer (approximately 11:00 a.m.). The results demonstrated that 9–10-cell embryos exhibiting an increase in blastomere number during this interval had significantly higher clinical pregnancy and live birth rates. Moreover, further analysis of all embryos within this window revealed that those with an increase in blastomere number achieved superior clinical pregnancy and live birth outcomes compared with those without such increases. These findings are consistent with the study by Lan et al. (23), who observed that frozen–thawed embryos showing an increase of ≥4 blastomeres during overnight culture exhibited significantly higher clinical pregnancy and implantation rates compared with embryos with no increase.

The potential mechanisms underlying the association between short-term blastomere increases and improved clinical outcomes remain incompletely understood. Previous studies suggested that differences in blastocyst formation rate and morphological quality may play important roles. Shapiro et al. reported that embryos reaching ≥9 cells at 72 hours post-retrieval were most likely to develop into expanded blastocysts, with a blastocyst formation rate as high as ~74% (14). In addition, variations in aneuploidy rates may also be critical. Pons et al. (19), in a retrospective analysis of 4,028 embryos, demonstrated that Day 3 embryos with fewer than eight cells were significantly less likely to develop into euploid blastocysts, whereas faster-cleaving embryos were more likely to achieve euploidy.

Taken together, these findings indicate that embryonic developmental potential cannot be fully assessed by static observations alone, and that short-term cleavage activity may serve as a more sensitive and precise biological marker of embryo quality. Clinically, when multiple embryos are eligible for transfer, short-term cleavage dynamics and changes in blastomere number may provide additional criteria for selection. Incorporating dynamic monitoring into embryo evaluation strategies offers a new dimension beyond traditional morphological assessment, potentially improving the accuracy and predictive power of current evaluation models. This approach may be particularly valuable for embryos classified as “intermediate quality” under conventional criteria, as the combination of static and short-term dynamic observations may yield a more reliable prediction of developmental potential.

This study also reaffirms the critical role of endometrial thickness in successful clinical pregnancy, consistent with prior studies (24–26). Optimal coordination between embryo quality and endometrial conditions evidently improves implantation success. Moreover, analysis of neonatal outcomes revealed no significant differences in gender, birth weight, or height among infants born from embryos of different blastomere count groups. This finding suggests that variations in early embryo cleavage rates primarily affect implantation potential and early pregnancy viability rather than long-term fetal growth and development. Thus, clinicians need not overly concern themselves with potential long-term developmental consequences of embryos showing short-term active cleavage.

Nonetheless, several limitations must be considered. First, the retrospective nature of this study carries inherent risks of selection bias, potentially affecting the generalizability of findings. The distribution of cycles across groups was uneven, particularly in the ≤7-cell group, where the sample size was relatively small and the proportion of older patients as well as those with premature ovarian insufficiency or diminished ovarian reserve was higher. This imbalance led to significant differences in some baseline variables. However, this reflects real-world clinical practice, in which 8-cell embryos are generally prioritized for transfer, and randomized controlled trials under such circumstances would be ethically impractical. Second, detailed morphological indicators such as embryo fragmentation, blastomere symmetry, multinucleation, and vacuole presence were not systematically evaluated. These factors, previously shown to be significant determinants of embryo implantation potential, should be incorporated into future assessments to comprehensively evaluate embryonic developmental potential. Lastly, as this was a single-center study with a limited sample size, larger-scale prospective multicenter studies are warranted to validate and enhance the reliability and clinical applicability of our findings.

In conclusion, our study demonstrates that both the number of blastomeres and their short-term dynamic changes prior to fresh SET significantly impact clinical pregnancy outcomes. Rapidly dividing embryos, particularly those with ≥11-cells, achieve superior clinical pregnancy and live birth outcomes. Additionally, short-term dynamic increases in blastomere number on the morning of embryo transfer correlate significantly with favorable clinical results. Therefore, integrating both static and dynamic blastomere observations into clinical embryo assessment protocols is recommended for more precise prediction of embryonic developmental potential.

## Data Availability

The raw data supporting the conclusions of this article will be made available by the authors, without undue reservation.

## Supplementary material

Supplementary Table 1 Pregnancy outcomes by embryo cell count in patients aged 35 years and younger.

Supplementary Table 2 Short-term blastomere increase and pregnancy outcomes.

Supplementary Table 3 Comparison of clinical outcomes between the 8-cell and >8-cell groups.
