# Supplementary material for: Association between pretransfer cleavage-stage blastomere dynamics and pregnancy outcomes in fresh single embryo transfer cycles: a retrospective cohort study
Source: Front Endocrinol (Lausanne). 2025 Sep 30;16:1672664. doi: 10.3389/fendo.2025.1672664 (PMC12518097; doi:10.3389/fendo.2025.1672664)
Supplement: Supplementary Table 1 — Pregnancy outcomes by embryo cell count in patients aged 35 years and younger. [file Table1.docx]

| Supplementary Table 1 Pregnancy outcomes by embryo cell count in patients aged 35 years and younger | | | | | |
| --- | --- | --- | --- | --- | --- |
| Variables | ≤7cell  n=30 | 8cell  n=197 | 9-10cell  n=107 | ≥11cell  n=115 | *P* |
|  |  |  |  |  |  |
| HCG positive rate , n (%) | 9 (30%) | 96 (48.7%) | 56 (52.3%) | 64 (55.7%) | 0.085 |
| Clinical pregnancy, n (%) | 5 (16.7%)^a^ | 76 (38.6%)^ab^ | 41 (38.3%)^ab^ | 57 (49.6%)^b^ | 0.009 |
| Early miscarriage, n (%) | -- | 10 (13.16%) | 6 (14.63%) | 4 (7.02%) | 0.049 |
| Live birth, n (%) | 2 (6.7%)^a^ | 59 (29.9%)^b^ | 34 (31.8%)^b^ | 48 (41.7%)^b^ | 0.003 |

Values are presented as number (percentage). P-values were calculated using χ² test. Post hoc pairwise comparisons were performed with Tukey’s test and Bonferroni adjustment. Within each row, values that do not share the same superscript letter (a, b) differ significantly (P ≤ 0.05).
