# Supplementary material for: Association between pretransfer cleavage-stage blastomere dynamics and pregnancy outcomes in fresh single embryo transfer cycles: a retrospective cohort study
Source: Front Endocrinol (Lausanne). 2025 Sep 30;16:1672664. doi: 10.3389/fendo.2025.1672664 (PMC12518097; doi:10.3389/fendo.2025.1672664)
Supplement: Supplementary Table 2 — Short-term blastomere increase and pregnancy outcomes. [file Table2.docx]

| Supplementary Table 2 Short-term blastomere increase and pregnancy outcomes | | | | |
| --- | --- | --- | --- | --- |
| Variables | No increase  n=350 | + 1–2 blastomeres  n=125 | + ≥3 blastomeres  n=86 | *P* |
|  |  |  |  |  |
| HCG positive rate, n (%) | 155 (44.25)^a^ | 73 (58.40)^b^ | 46 (53.48)^ab^ | 0.016 |
| Clinical pregnancy, n (%) | 115 (32.86)^a^ | 58 (46.40)^b^ | 41 (47.67)^b^ | 0.004 |
| Early miscarriage, n (%) | 18 (15.65) | 9 (15.52) | 3 (7.32) | 0.485 |
| Live birth, n (%) | 87 (24.86)^a^ | 48 (38.4)^b^ | 33 (38.37)^b^ | 0.003 |

Values are presented as number (percentage). P-values were calculated using χ² test. Post hoc pairwise comparisons were performed with Tukey’s test and Bonferroni adjustment. Within each row, values that do not share the same superscript letter (a, b) differ significantly (P ≤ 0.05).
