# Supplementary material for: Association between pretransfer cleavage-stage blastomere dynamics and pregnancy outcomes in fresh single embryo transfer cycles: a retrospective cohort study
Source: Front Endocrinol (Lausanne). 2025 Sep 30;16:1672664. doi: 10.3389/fendo.2025.1672664 (PMC12518097; doi:10.3389/fendo.2025.1672664)
Supplement: Supplementary Table 3 — Comparison of clinical outcomes between the 8-cell and >8-cell groups. [file Table3.docx]

| Supplementary Table 3. Comparison of clinical outcomes between the 8-cell and >8-cell groups | | | |
| --- | --- | --- | --- |
| Variables | 8cell  n=248 | ＞8cell  n=266 | *P* |
|  |  |  |  |
| HCG positive rate, n (%) | 118 (47.58) | 144 (54.14) | 0.162 |
| Clinical pregnancy, n (%) | 91 (36.69) | 118 (44.36) | 0.093 |
| Early miscarriage, n (%) | 15 (16.48) | 13 (11.02) | 0.307 |
| Live birth, n (%) | 69 (27.82) | 97 (36.47) | 0.046 |
